# Disturbed circadian rhythm and retinal degeneration in a mouse model of Alzheimer’s disease

**DOI:** 10.1186/s40478-023-01529-6

**Published:** 2023-03-31

**Authors:** Laura Carrero, Desireé Antequera, Ignacio Alcalde, Diego Megías, Joana Figueiro-Silva, Jesús Merayo-Lloves, Cristina Municio, Eva Carro

**Affiliations:** 1 Group of Neurodegenerative Diseases, Hospital Universitario 12 de Octubre Research Institute (imas12), 28041 Madrid, Spain; 2 Network Center for Biomedical Research in Neurodegenerative Diseases (CIBERNED), ISCIII, Madrid, Spain; 3 Autonoma de Madrid University, Madrid, Spain; 4 Neurobiology of Alzheimer’s Disease Unit, Functional Unit for Research into Chronic Diseases, Instituto de Salud Carlos III, Madrid, Spain; 5 Instituto Universitario Fernández-Vega, Universidad de Oviedo and Fundación de Investigación Oftalmológica, Oviedo, Spain; 6 Instituto de Investigación Sanitaria del Principado de Asturias (ISPA), Oviedo, Spain; 7 Advanced Optical Microscopy Unit, Unidades Centrales Científico-Técnicas, Instituto de Salud Carlos III, Madrid, Spain; 8 Institute of Medical Genetics, University of Zurich, Zurich, Switzerland; 9 Department of Molecular Life Science, University of Zurich, Zurich, Switzerland

**Keywords:** Alzheimer’s disease, Circadian rhythm, Clock genes, Retina, Transgenic mice, Retinal ganglion cells, Retinohypothalamic tract, Hypothalamus, Amyloid, Melanopsin

## Abstract

The circadian clock is synchronized to the 24 h day by environmental light which is transmitted from the retina to the suprachiasmatic nucleus (SCN) primarily via the retinohypothalamic tract (RHT). Circadian rhythm abnormalities have been reported in neurodegenerative disorders such as Alzheimer's disease (AD). Whether these AD-related changes are a result of the altered clock gene expression, retina degeneration, including the dysfunction in RHT transmission, loss of retinal ganglion cells and its electrophysiological capabilities, or a combination of all of these pathological mechanisms, is not known. Here, we evaluated transgenic APP/PS1 mouse model of AD and wild-type mice at 6- and 12-month-old, as early and late pathological stage, respectively. We noticed the alteration of circadian clock gene expression not only in the hypothalamus but also in two extra-hypothalamic brain regions, cerebral cortex and hippocampus, in APP/PS1 mice. These alterations were observed in 6-month-old transgenic mice and were exacerbated at 12 months of age. This could be explained by the reduced RHT projections in the SCN of APP/PS1 mice, correlating with downregulation of hypothalamic GABAergic response in APP/PS1 mice in advanced stage of pathology. Importantly, we also report retinal degeneration in APP/PS1 mice, including Aβ deposits and reduced choline acetyltransferase levels, loss of melanopsin retinal ganglion cells and functional integrity mainly of inner retina layers. Our findings support the theory that retinal degeneration constitutes an early pathological event that directly affects the control of circadian rhythm in AD.

**Supplementary Information:**

The online version contains supplementary material available at 10.1186/s40478-023-01529-6.

## Introduction

Alzheimer’s disease (AD), the most prevalent form of dementia worldwide [8], is characterized by aggregation and accumulation of amyloid-β (Aβ), and hyperphosphorylated tau protein in the patients’ brains. As well as deteriorating cognitive functions [5, 47, 62], patients with AD show circadian dysfunctions, even at preclinical stages, that may contribute to disease severity [53, 69].

Circadian rhythm dysfunction is markedly associated with aging [33] but is aggravated in patients with neurodegenerative diseases, including AD [36, 42, 53]. Patients with AD also have functional and morphological alterations not only in the “master clock” of the circadian network, the suprachiasmatic nucleus (SCN) in the hypothalamus [67], but also in the cerebral cortex associated with disrupted rhythms [43], and in the hippocampus associated with synaptic activity and cognition [24]. However, although numerous studies, including those developed in mouse models of AD, have examined circadian changes, the underlying mechanisms leading this circadian dysfunction are not well understood.

A circadian rhythm is an approximately 24-h cycle in the physiological processes of most organisms that is endogenously generated and can be modulated by external cues. Circadian cycles show rhythmicity, as they persist with a cycle of approximately 24 h. The circadian rhythmicity is typically measured by three parameters: amplitude, phase and period. Amplitude is defined as the magnitude of a cycle, or the difference between the peak and trough values. Phase indicates the time of day of peak activity, averaged over several days, is defined as the timing of a reference point in the cycle relative to a fixed event. And period is the length of time of a full circadian cycle, between two reference points within a rhythm or recurring wave [7, 42]. Circadian rhythms in behavior are largely driven by the activity of the SCN. At a molecular level, circadian rhythms are driven by oscillations of circadian clock genes in the SCN and other brain tissues. These circadian clock genes include *BMAL1* and *CLOCK*, which function as transcription factors to drive transcription of their own repressors: *PER1*, *PER2*, *PER3*, *CRY1*, and *CRY2*. Levels of these clock gene transcripts oscillate with a 24-h period in most tissue and are entrained to daily light cycles [13]. Thus, measurement of clock gene oscillations in SCN and other tissues can be a molecular marker of clock function.

Circadian rhythms are regulated by a system that includes the retina, and the hypothalamus, connected via the retinohypothalamic tract (RHT) [27]. In AD, thinning of the retinal nerve fiber layer due to the selective death of retinal ganglion cells (RGCs), and melanopsin RGCs loss have been reported [4, 9, 38, 40]. RGC degeneration was also described in several mouse models of AD [16, 68]. The RHT, originates from the RGCs, appears to be the most important pathway for communicating photic information to the SCN because its destruction eliminates the ability of an animal to entrain to the light–dark cycle [35]. A small percentage of RGCs project light signals through the RTH to the SCN due to the expression of melanopsin the photopigment responsible for this intrinsic photosensitivity (mRGCs) [10, 27, 29]. Although morphological and functional abnormalities were reported in optic nerve in AD [32, 51], few studies have focused on the RHT in AD.

Stimulation of the SCN via RHT glutamatergic projections, initiates a cascade of transcriptional–translational feedback loops that eventually lead circadian rhythms. γ-aminobutyric acid (GABA), the major inhibitory neurotransmitter in the mature central nervous system (CNS) [57], is synthesized through the decarboxylation of glutamate by the enzyme glutamic acid decarboxylase (GAD) [12]. It has been proposed that glutamate release induced by light stimulates GABA release in the SCN inducing a phase delay in the circadian pacemaker [1].

AD pathology also manifests itself in retina, with morphological and functional disturbances [26]. Retina is enriched with neurons including RGCs, photoreceptor cells, bipolar cells, amacrine cells, and horizontal cells [34]. Aβ deposition in the retina is neurotoxic and potentially fatal to RGCs [19, 40, 61]. Loss of mRGCs has been described in AD suggesting that this retinal alteration could contribute to circadian dysfunction [40]. Meanwhile, retinal cell functioning can be disrupted by defects in neurotransmitters such as acetylcholine (ACh) [55].

In the present study we used APP/PS1 mice as an AD animal model, to demonstrate that retinal defects lead to impairment in information projection via RHT to the SNC, therefore leading to disruption of the circadian cycle. Here, we studied in depth the structural pathway by which light information entrainment of the circadian clock in the hypothalamus from the retina and through the RTH.

## Material and methods

### Animals

Male double transgenic APP/PS1 mice (6- and 12-month-old), a cross between Tg2576 (overexpressing human APP695) and mutant PS1 (M146L), were used from our inbred colony (Instituto de Investigacion Hospital 12 de Octubre). Age-matched littermate mice not expressing the transgene were used as wild-type controls (wt), with the same C57/Bl6 background than the transgenic mice. Animals were sacrificed by deep anaesthesia and perfused transcardially either with saline for biochemical analysis, or 4% paraformaldehyde (PFA) in 0.1 M phosphate buffer (PB), pH 7.4 for immunohistochemical analysis. All animals were handled and cared for according to the Council Directive 2010/63/UE of 22 September 2010, the ARVO Statement for the Use of Animals in Ophthalmic and Vision Research and ARRIVE guidelines (2020), and procedures were approved by the Hospital 12 de Octubre Ethics Committee. Mice were maintained on a 12:12 light/dark cycle (Zeitgeber time (ZT)) with ad libitum access to food and water and sacrificed at ZT 1 (08:00), 7 (14:00), 13 (20:00) and 19 (02:00).

### Anterograde labeling of retinal hypothalamic tracts

The retinal hypothalamic tracts (RHTs) of APP/PS1 and wt mice were labeled at 6 and 12 months of age with Alexa Fluor 488-conjugated cholera toxin B (CTB). Each mouse was anesthetized with isofluorane and slowly injected into one vitreous chamber with a 10 μl syringe (Hamilton 701LT, 10 µl). 6 µl of Alexa Fluor 488-conjugated CTB was slowly and unilaterally injected into the eye. Seven days after the injection, mice were sacrificed by deep anaesthesia and perfused transcardially with 4% PFA.

### Electroretinography

Animals were dark adapted overnight prior to electroretinography (ERG) recordings and were handled under dim red light. Mice were anaesthetized with a mixture of ketamine hydrochloride (80 mg/kg; Imalgene 1000, Merial Laboratorios S.A., Barcelona, Spain) and xylazine hydrochloride (5 mg/kg; Rompun, Bayer Hispania S.L., Barcelona, Spain) and bilateral pupil dilatation was induced by applying in both eyes a topical drop of 1% Tropicamide (Alcon Cusí, SA, Barcelona, Spain). A drop of Hydroxypropyl methylcellulose (2% Methocel, OmniVision GmbH, Puchheim, Germany) was placed between the eye and the electrode to facilitate conductivity.

ERG RETI-Animal Port-Scan21 equipped with a Ganzfeld Q450 C sphere (Roland Consult, Germany) was used to quantify retinal function under the ISCEV protocol including scotopic responses (step 1: − 25 dB, 0.0095 cds/m^2^, 0.476 Hz; step 2: 0 dB, 3.00 cds/m^2^, 0.095 Hz; step 3: Oscillatory potentials, 0 dB, 3.00 cds/m^2^, 0.095 Hz); and photopic responses after 10 min of light adaptation (step 4: 5 dB, 9.49 cds/m^2^, 0.625 Hz; step 5: 0 dB, 3.00 cds/m^2^, 29.412 Hz). A minimum of 10 flashes were recorded and averaged at each step. Electrode impedance was accepted with a difference of < 9 KΩ between electrodes. The amplitude of the a-wave (measured from baseline to peak) and b-wave (measured from the a-wave through to the b-wave peak) were analyzed. Recordings were performed at 6 and 12 months of age of the same APP/PS1 (n = 9) and wt (n = 6) mice.

### ELISA assays

ELISA was performed for quantification of circadian rhythm proteins (CLOCK, ARNTL, PER2 and CRY1) in the hippocampus, hypothalamus and cortex of 6- and 12-month-old APP/PS1 and wt mice. Mouse-specific commercial kits used were: CLOCK ELISA Kit (SEQ116Mu, Cloud-Clone Corp), ARNTL ELISA Kit (SED468Mu Cloud-Clone Corp), CRY1 ELISA Kit (E-EL-M0364, Elabscience) and PER2 ELISA Kit (MBS9909846 MyBioSource). In all cases, tissue samples were diluted in PB saline (PBS) and loaded in duplicate, following the manufacturer’s instructions. Protein concentrations were measured using a BCA assay (Pierce BCA Protein Assay Kit, Thermo Fisher, Waltham, MA, USA).

### RNA extraction and quantification

At 6 and 12 months of age, mice were euthanized via CO_2_ inhalation at ZT 1, 7 13 and 19. Brains were immediately removed and cerebral cortex, hippocampus and hypothalamus were extracted and stored at − 80 °C. RNA was obtained from APP/PS1 and wt brain tissues (cerebral cortex, hippocampus and hypothalamus) using NZYol (NZYTech, Lda., Lisboa, Portugal) following the manufacturer's protocol. RNA concentration was measured in a NanoDrop™ One Spectrophotometer (ThermoFisher) and 1 µg of each sample was retrotranscribed to cDNA using iScript™ cDNA Synthesis Kit (Bio-Rad). Quantitative real-time PCR (qRT-PCR) was performed in a LightCycler® 480 Instrument (Roche Diagnostics) using NZYSpeedy qPCR Green Master Mix (NZYTech, Lda., Lisbon, Portugal). The primers were predesigned and used in the qRT-PCR to determine the expression levels of *Clock*, *Per*, *Cry*, *Arntl*, and the housekeeping gene (*Hprt*) (Additional file 1: Table S1). Relative levels of mRNA were calculated using crossing-point (Cp) data and ΔΔCp method (also known as ΔΔCt). Cp data from the gene of interest (GOI) were normalised to mean of endogenous gene *HPRT* data to obtain ΔCp data (ΔCp = mean Cp_*Hprt*_—Cp_*GOI*_). ΔΔCp was calculated between the normalised ΔCp values from each time point.

### Immunoblotting

The proteins were obtained from the organic phase of the RNA extraction process, following the manufacturer’s instructions. Subsequently, 500 μl of a lysis buffer composed of 0.5 M EDTA, 1 M NaCl, 10% SDS, Tris pH8, protease and phosphatase inhibitors. Afterwards, it was incubated in a thermoblock (Eppendorf) for 2 h at 50 °C, after which it was sonicated at intensity 3 for 10 s. Finally, it was centrifuged at 10,000 g for 10 min.

The supernatant was recovered and stored at − 80 °C. Protein estimation was determined using the BCA assay (Pierce BCA Protein Assay Kit, Thermo Fisher, Waltham, MA, USA). Each sample was loaded in a precast 10% Tris–HCl (CriterionTM TGX Stain-FreeTM Precast Gels, BioRad Laboratories, CA, USA) and the separated proteins were transferred to nitrocellulose membranes (BioRad Laboratories). Primary antibody utilised was: mouse monoclonal anti- GAD67 (MAB5406, Merck Millipore, MA, USA) Protein loading was monitored using a mouse monoclonal antibody against β-actin (A1978, Sigma-Aldrich, St. Louis, USA). Membranes were then incubated for 1 h with the appropriate horseradish peroxidase-conjugated secondary antibodies (Dako, CA, USA), and immunocomplexes were revealed by an enhanced chemiluminescence reagent (ECL Clarity; BioRad Laboratories). Densitometric quantification was carried out with Image Studio Lite 5.0 software (Li-COR Biosciences, NE, USA). Protein bands were normalised to β-actin levels and expressed as a percentage of the control group.

### Immunohistochemistry

Fixed brains and eyes were cut on a vibratome (Leica Microsystems) and a cryostat Microm HM550 (Thermo Scientific, Waltham, MA, USA) at 30 μm and 7 µm, respectively.

To carry out immunohistochemistry for Aβ deposits, brain slices were pre-incubated for 20 min with 88% formic acid at room temperature and immunolabeled with mouse anti-Aβ antibody (1:500, MBL M046-3, Nagoya, Japan) at 4 °C diluted in PBS 0.1 M containing 5% normal goat serum and 0.5% Triton X-100. After overnight incubation, primary antibody staining was revealed using the avidin–biotin complex method (VECTASTAIN Elite ABC Kit, Vector Laboratories, Burlingame, CA) and 3,3′-diaminobenzidine chromogenic reaction (Vector Laboratories, Inc). Finally, the slices were mounted with DPX (Panreac). Images were captured using a light microscope (Zeiss microscope; Carl Zeiss Microimaging, GmbH, Oberkochen, Germany).

For retinal flat mount immunostaining, globes were enucleated and fixed in phosphate buffer 4% PFA. Retinas were carefully separated from the globe, cuts were made in the four quadrants (superior, inferior, nasal and temporal) and retinal flat mounts were prepared. For immunofluorescence, the retinal flat mounts, brain tissue and eye slices were incubated overnight with primary antibodies at 4 °C diluted in PBS 0.1 M containing 5% normal horse serum and 0.3% Triton X-100 or10% normal horse serum and 0.03% Triton X-100, respectively. One series of sections was used for double-labelling experiments and incubated with the corresponding primary antibodies which are as follows: rabbit anti-NeuN (1:500, Merck Millipore ABN78, MA, USA), mouse anti-β-amyloid (1:500, MBL M046-3, Nagoya, Japan), rabbit anti-CTβ (1:200, Abcam AB-34992, Cambridge, UK), mouse anti-GAD67 (1:1000, Merck Millipore MAB-5406, MA, USA), goat anti-ChAT (1:200, Merck Millipore AB144P, MA, USA), rabbit anti Opn4 (1:1000, ATS (Iggy)AB-N38, CA, USA). These antibodies were revealed using fluorescence-conjugated secondary antibodies from Life Technologies: Alexa Donkey anti-mouse 488 (A21202, Molecular Probes) Alexa Goat anti-rabbit 555 (A27039, Molecular Probes), Alexa Donkey anti-goat 488 (A11055, Molecular Probes) and FITC goat anti-rabbit (Santa Cruz Biotechnology, Inc., SC-2012, TX, USA). Finally, the slices were mounted with Immunoselect Antifading Mounting Medium with DAPI (SCR-038448, BioTrend). Fluorescent images were obtained with a Stellaris Laser Scanning confocal microscope or a Thunder imager wide-field microscope (Leica Microsystems), and analysed using the Volocity 3D Image Analysis program.

### Statistical analysis

Data analysis was conducted using GraphPad Prism 6.01 (GraphPad Software, USA) software. All data are expressed as mean ± standard error of the mean (SEM). For multiple comparisons was calculated by two-way ANOVA followed by Bonferroni’s correction. In all cases, statistical significance was set at *p* < 0.05. For the analysis of circadian rhythmicity, we used CircaCompare in RStudio software (version 1.1.419), as previously described [60].

## Results

### Alterations to the molecular circadian clock in the hypothalamus of APP/PS1 mice

**Fig. 1:**
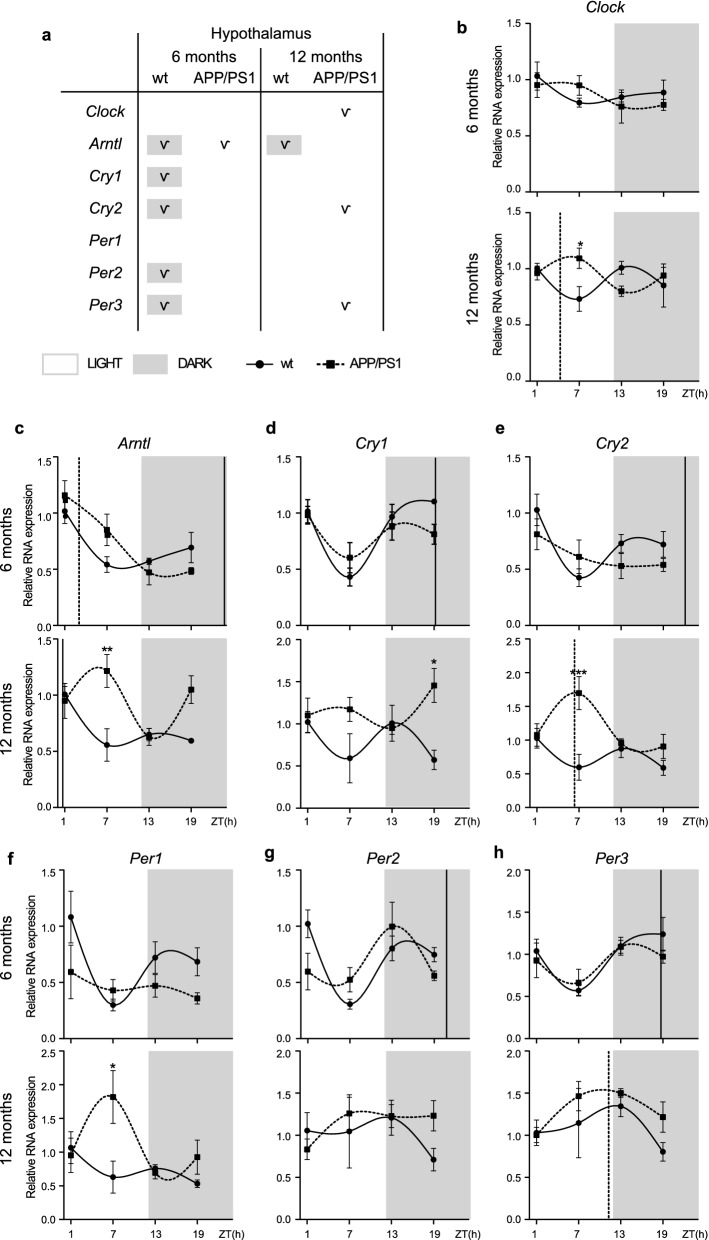
Altered expression profile of clock genes in the hypothalamus of APP/PS1 mice. **a** Schematic table symbolizing the circadian rhythmicity of clock genes, indicating diurnal (light) or nocturnal (dark) acrophase, in the studied mice groups. **b**–**h** Transcript levels from wt (filled circles) and APP/PS1 mice (filled squares) 6- and 12-month-old mice at ZT 1, 7, 13, and 19 for **b** *Clock*, **c** *Arntl*, **d** *Cry1*, **e** *Cry2*, **f** *Per1*, **g** *Per2*, and **h** *Per3*. Dashed lines indicate the time of acrophase for each mice group. 4–5 mice per time point and group were analyzed. Mean ± SEM. *p < 0.05, **p < 0.01, ***p < 0.001 using two-way ANOVA and Bonferroni’s multiple comparison post-test. *wt* wild type, *ZT* Zeitgeber Time

Since the major circadian pacemaker in mammals is located in the SCN, we first determined whether the variations in clock genes expression were observed in the 6- and 12-month-old APP/PS1 and wt mice littermates, referred as the initial and advance/severe pathology state, respectively (Fig. 1). To test this hypothesis, we extracted whole hypothalamus from these mice groups and performed qRT-PCR to examine mRNA expression of *Clock, Arntl, Cry1, Cry2, Per1, Per2* and *Per3* at ZT 1 (08:00), 7 (14:00), 13 (20:00) and 19 (02:00). We analyzed rhythmic expression pattern of these clock gene in APP/PS1 mice compared with wt mice.

At 6 months, *Clock* mRNA expression is constant and independent of the light cycle, but at 12 months, it begins to be affected by light, demonstrating a certain degree of rhythmicity, being statistically significant in APP/PS1 mice, according to the analysis with the software (Fig. 1a, b; Additional file 2: Table S2). *Arntl* transcription showed a rhythmic pattern in wt mice of both ages with a peak (acrophase) during dark period (Fig. 1a, c; Additional file 2: Table S2). Although the oscillatory expression of *Arntl* was kept in 6-month-old APP/PS1 mice, our findings revealed a delayed expression acrophase of *Arntl* mRNA, appearing during light phase, in AD transgenic mice compared with wt mice (Fig. 1a, c; Additional file 2: Table S2). However, one of the most remarkable result was the disruption of circadian rhythmicity of negative regulators *Cry1, Cry2, Per2*, and *Per3* in 6-month-old APP/PS1 mice. *Cry1, Cry2, Per2*, and *Per3* mRNA expression showed a rhythmic expression pattern, including nocturnal acrophase, only in 6-month-old wt mice, while this oscillation was absent in age-matched APP/PS1 mice (Fig. 1a, d, e, g, h; Additional file 2: Table S2).

With aging, *Cry1, Cry2, Per2,* and *Per3* oscillation was lost in wt mice, meanwhile in 12-month-old APP/PS1 mice circadian rhythmicity of *Cry2,* and *Per3* was preserved but with a diurnal acrophase (Fig. 1a, e, h; Additional file 2: Table S2).

### Alterations to the molecular circadian clock in the hippocampus of APP/PS1 mice

**Fig. 2:**
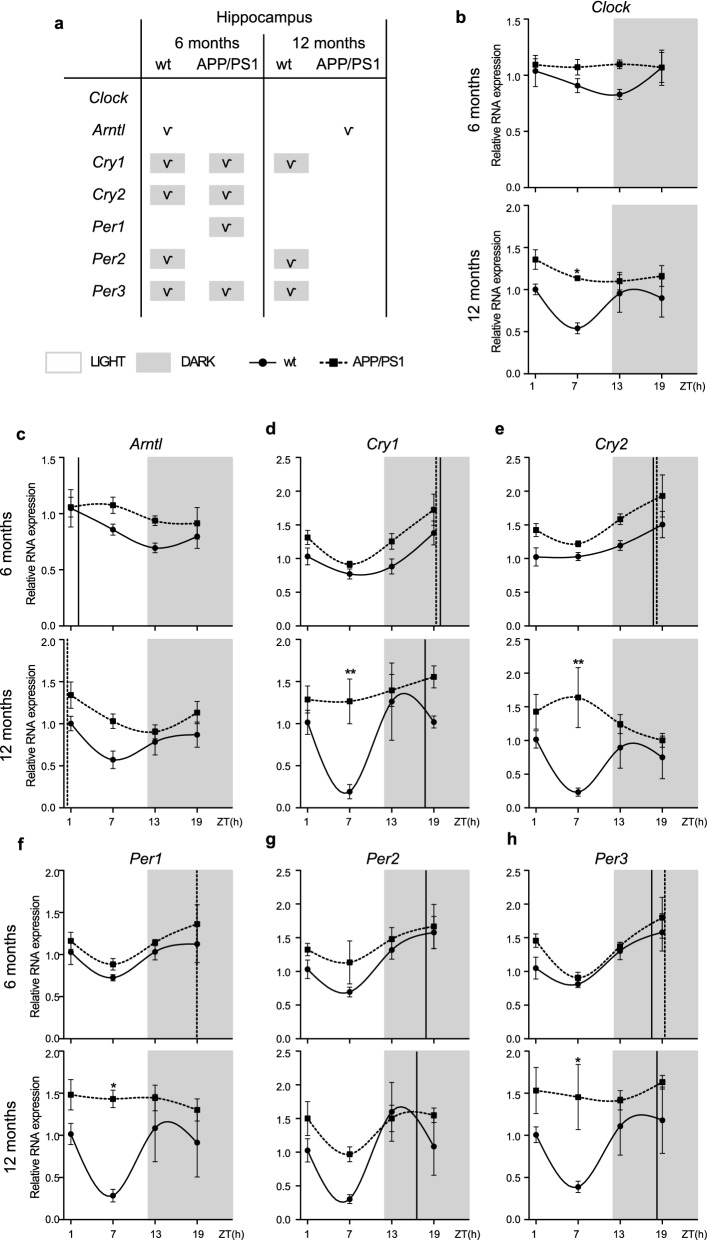
Altered expression profile of clock genes in the hippocampus of APP/PS1 mice. **a** Schematic table symbolizing the circadian rhythmicity of clock genes, indicating diurnal (light) or nocturnal (dark) acrophase, in the studied mice groups. **b**–**h** Transcript levels from wt (filled circles) and APP/PS1 mice (filled squares) 6- and 12-month-old mice at ZT 1, 7, 13, and 19 for **b** *Clock*, **c** *Arntl*, **d** *Cry1*, **e** *Cry2*, **f** *Per1*, **g** *Per2*, and **h** *Per3*. Dashed lines indicate the time of acrophase for each mice group. 4–5 mice per time point and group were analyzed. Mean ± SEM. *p < 0.05, **p < 0.01 using two-way ANOVA and Bonferroni’s multiple comparison post-test. *wt* wild type, *ZT* Zeitgeber Time

Circadian clock is present both in the SCN of the hypothalamus as well as in peripheral tissues [28] where it drives rhythms in cellular processes in part by modulating the abundance of gene transcripts involved in these processes. One of the main brain areas affected by AD pathology is the hippocampus, and alterations in the expression of clock genes in this specific area might contribute to the neurodegenerative disease. Thus, we examined mRNA expression of the above comment clock genes in hippocampal tissue from 6- and 12-month-old APP/PS1 and wt mice. We found that the expression of clock genes mRNA in the hippocampus of APP/PS1 mice was significantly altered and showed abnormal circadian oscillations compared with those of wt mice (Fig. 2).

*Clock* mRNA expression didn’t show circadian rhythm in any of the mice groups (Fig. 2a, b; Additional file 3: Table S3). *Arntl* mRNA expression showed a rhythmic pattern in 6-month-old wt mice with an acrophase at light period, whereas age-matched APP/PS1 mice lost this oscillation (Fig. 2a, c; Additional file 3). With aging, *Arntl* mRNA showed a rhythmic expression only in APP/PS1 mice, with an acrophase in the transition from dark to light (Fig. 2a, c; Additional file 3: Table S3). Meanwhile negative regulators *Cry1, Per2*, and *Per3* exhibit a rhythmic expression pattern in 12-month-old wt mice, APP/PS1 mice lost this rhythmic expression at 12 months of age (Fig. 2a, d, g, h; Additional file 3: Table S3).

### Alterations to the molecular circadian clock in the cerebral cortex of APP/PS1 mice

There is particular interest in characterizing the rhythmicity of clock genes in cerebral cortex because there are prominent daily rhythms in soluble Aβ levels [37], and rhythms of gene expression in cerebral cortex could be a mechanism contributing to this effect. Thus, we examined mRNA expression of the above comment clock genes in the cerebral cortex of 6- and 12-month-old APP/PS1 and wt mice, finding significant alteration.

**Fig. 3:**
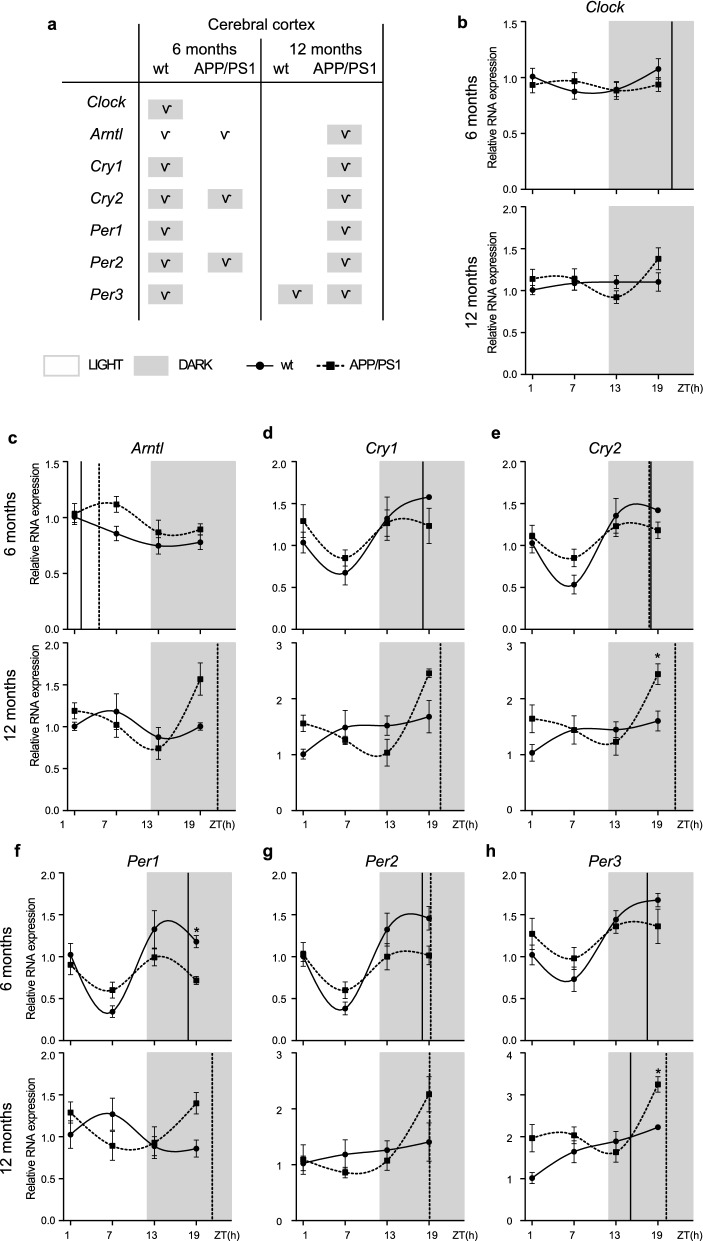
Altered expression profile of clock genes in the cerebral cortex of APP/PS1 mice. **a** Schematic table symbolizing the circadian rhythmicity of clock genes, indicating diurnal (light) or nocturnal (dark) acrophase, in the studied mice groups. **b**–**h** Transcript levels from wt (filled circles) and APP/PS1 mice (filled squares) 6- and 12-month-old mice at ZT 1, 7, 13, and 19 for **b** *Clock*, **c** *Arntl*, **d** *Cry1*, **e** *Cry2*, **f** *Per1*, **g** *Per2*, and **h** *Per3*. Dashed lines indicate the time of acrophase for each mice group. 4–5 mice per time point and group were analyzed. Mean ± SEM. *p < 0.05 using two-way ANOVA and Bonferroni’s multiple comparison post-test. *wt* wild type, *ZT* Zeitgeber Time

*Clock* expression show significant oscillation with an acrophase during dark period (at ~ 21:00 h) only in 6-month-old wt mice (Fig. 3a, b; Additional file 4: Table S4). *Arntl* mRNA expression showed a rhythmic pattern in 6-month-old wt mice with an acrophase at light period (Fig. 3a, c; Additional file 4: Table S4). Although the oscillatory expression of *Arntl* was kept in age-matched APP/PS1 mice, acrophase was delayed in these AD transgenic mice compared with control mice (Fig. 3a, c; Additional file 4: Table S4).

With aging, rhythmic expression of *Arntl*, *Cry1, Cry2 Per1,* and *Per2* was lost in wt mice, meanwhile in 12-month-old APP/PS1 mice circadian rhythmicity of these negative regulator genes was preserved as well as an acrophase of *Arntl* mRNA also at dark period (Fig. 3a, c–g; Additional file 4: Table S4).

**Fig. 4:**
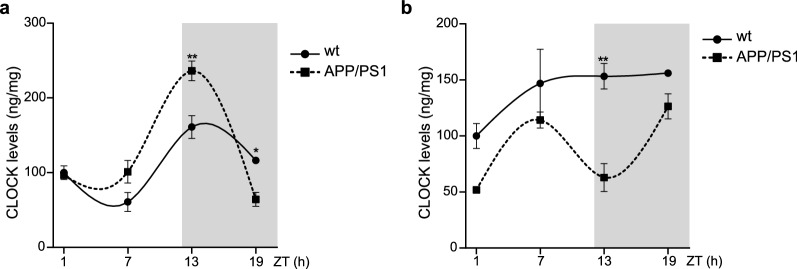
Altered expression profile of CLOCK proteins of APP/PS1 mice. **a**, **b** Quantification of CLOCK proteins levels in **a** hippocampus and **b** cerebral cortex from APP/PS1 at 12-month-old mice at ZT1, ZT7, ZT13 and ZT19 groups is shown (n = 3–5 mice per group). Data are expressed as mean ± SEM. *p < 0.05; **p < 0.01; using two-way ANOVA and Bonferroni’s multiple comparison post-test. *wt* wild type

To test if these changes in circadian clock gene mRNA expression also manifested at the protein level, we assessed the levels of proteins encoded by these clock genes (Fig. 4). Meaningfully, we found a clear relation between CLOCK levels and gene expression in the hippocampus and cerebral cortex in 12-month-old mice. In the hippocampus, CLOCK levels were higher in APP/PS1 mice compared with APP/PS1 mice during light phase, and diminished during dark period (Fig. 4a), in a similar manner than that observed regards the *Clock* mRNA expression (Fig. 2b). At cortical level, we found that Clock levels were lower at ~ 13ZT period in APP/PS1 mice compared with age-matched wt mice (Fig. 4b), accordingly with the expression pattern of *Clock* mRNA previously shown (Fig. 3b).

### Amyloid neuropathology in the brain of APP/PS1 mice

**Fig. 5:**
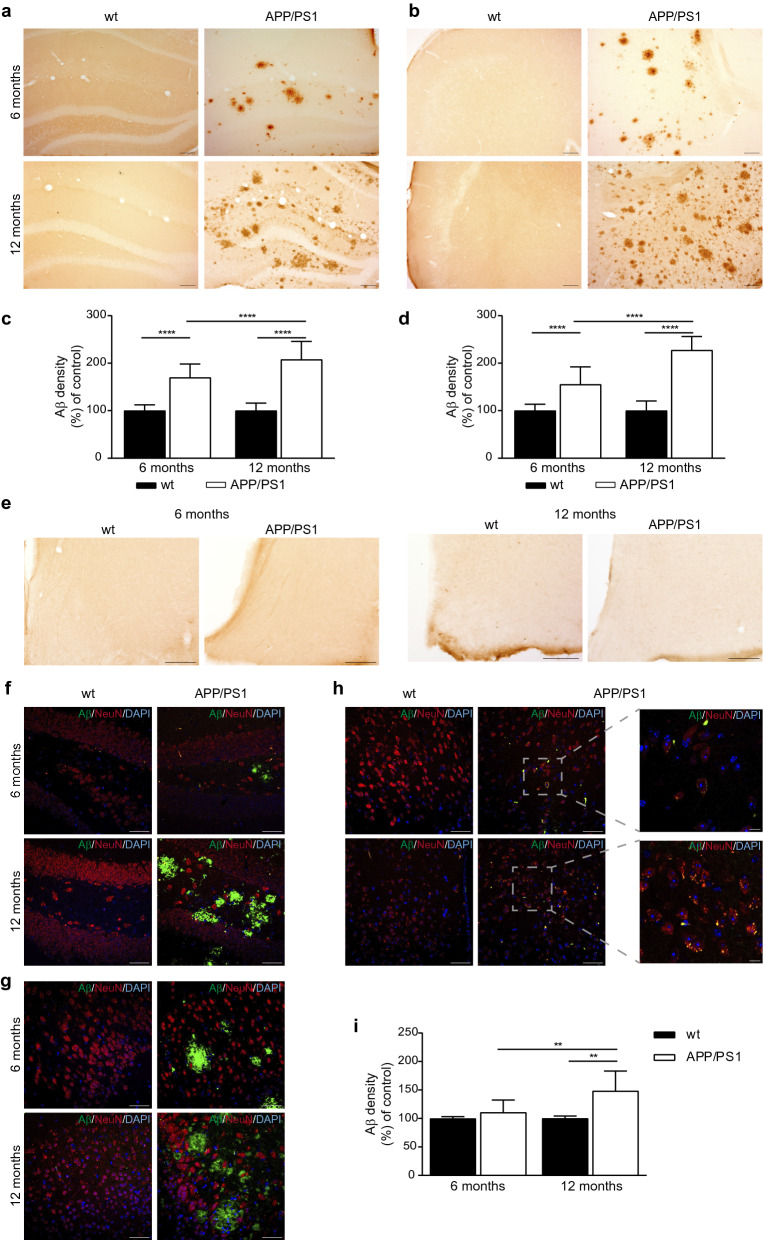
Brain Aβ deposition in APP/PS1 mice. **a**, **b** Representative images showing Aβ deposits in the **a** hippocampus and **b** cerebral frontal cortex in APP/Ps1 mice are increased with aging (scale bar 10 µm). **c**, **d** Quantification of Aβ burden in **c** hippocampus and **d** cerebral cortex from the APP/PS1 mice groups is shown (n = 2–6 mice per group). **e** Representative images showing the lack of Aβ deposits in the hypothalamic area (scale bar 10 µm). **f**–**h** Representative confocal images showing Aβ deposits (green) and neurons immunoslabelled with anti-NeuN (red) in the **f** hippocampus, **g** cerebral frontal cortex, and **h** hypothalamus in APP/PS1 mice (scale bar 50 µm) Inserts with higher magnification showing Aβ accumulated in NeuN^+^ hypothalamic cells (scale bar 10 µm). Nuclei are stained with DAPI (blue). **i** Quantitative analysis of the fluorescence intensity of Aβ staining in hypothalamic neurons showing a significant increase in 6- and 12-month-old APP/PS1 mice (n = 2–6 mice per group). Data are expressed as mean ± SD. **p < 0.01; ***p < 0.001; ****p < 0.0001 using two-way ANOVA and Bonferroni’s multiple comparison post-test. *wt* wild type

To verify whether variations in the clock pathway were related to brain pathology in APP/PS1 mice, immunohistochemical analysis of Aβ accumulation was performed in the brain areas where the clock genes expression was investigated. As expected and shown in Fig. 5, 6-month-old APP/PS1 mice showed a significant increase in Aβ immunostaining compared to age-matched wt mice in both hippocampus and cerebral cortex, whereas no Aβ immunostaining was detected in hypothalamic areas of APP/PS1 mice by immunohistochemistry assay (Fig. 5a, b, e). Moreover, the presence of Aβ plaques present in 12-month-old APP/PS1 mouse group was higher in both brain areas (Fig. 5a, b). Quantitative analysis of brain amyloid staining, expressed as the percentage of brain parenchyma stained with Aβ, confirmed these effects in cortical and hippocampal areas (Fig. 5c, d). We also used double staining with anti-NeuN and anti-Aβ antibodies to detect amyloid deposits, and confirmed the escalation in amyloid deposits in hippocampus and cerebral cortex in APP/PS1 mice by immunofluorescence (Fig. 5f, g). However, when fluorescence immunohistochemistry and confocal image acquisition was conducted using double staining with anti-NeuN and anti-Aβ antibodies, we were able to detect intracellular Aβ immunostaining within neurons in hypothalamus (Fig. 5h), and quantitative analysis revealed a significant increase in Aβ deposition in 12 month-old APP/PS1 mice in hypothalamic neurons (Fig. 5i).

### Abnormal RHT projection in the hypothalamus of APP/PS1 mice

**Fig. 6:**
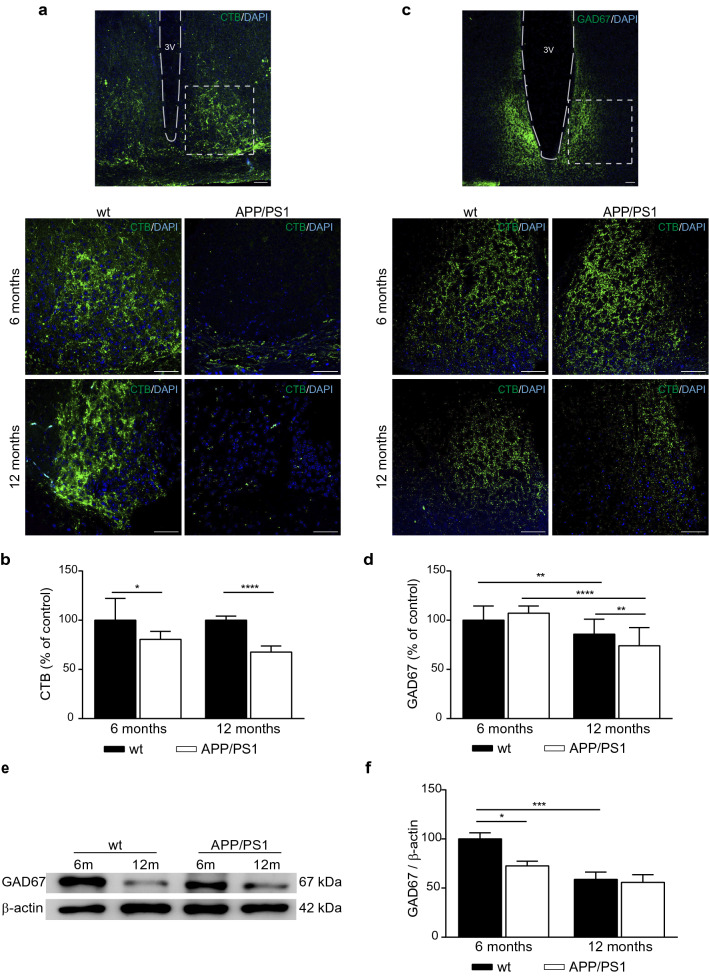
CTB-labeled retinohypothalamic tract projections and GAD67 expression in the hypothalamus of APP/PS1 mice. **a** Upper panel showing the periventricular area at low magnification; botom panels showing at higher magnification representative fluorescence images of RHT projections after CTB injections into the eyes of 6- and 12-month-old wt and APP/PS1 mice. Nuclei are stained with DAPI (blue) (scale bar 50 µm). **b** Quantitative analysis of the fluorescence intensity of CTB labeling in SCN revealed a significant reduction of this intensity in 6- and 12-month-old APP/PS1 mice compared with age-matched control mice (n = 5 mice per group). **c** Upper panel showing the periventricular area at low magnification; botom panels showing representative fluorescence images of GAD67 in 6- and 12-month-old wt and APP/PS1 mice (scale bar 50 µm). **d** Quantitative analysis of the fluorescence intensity of GAD67 labeling in SCN showed a significant reduction with aging and with the genotype and 12 months of age (n = 3–4 mice per group). Data are expressed as mean ± SD. **e** The levels of GAD67 in SCN from 6- to 12-month-old wt and APP/PS1 mice were evaluated by western blot and representative immunoblots shown. **f** Histograms representing the protein densitometric analysis are presented (n = 4–9 mice per group). Data are expressed as mean ± SEM; *p < 0.05; **p < 0.01; ***p < 0.001; ****p < 0.0001 using two-way ANOVA and Bonferroni’s multiple comparison post-test. *CTB* cholera toxin B, *RHT* retinohypothalamic tract, *SCN* suprachiasmatic nucleus, *wt* wild type

The RHT appears to be the most important pathway for communicating photic information to the SCN because its destruction eliminates the ability of an animal to entrain to the light–dark cycle [35]. To ascertain whether circadian clock alterations of APP/PS1 mice is due to a disruption or an absence of an entrainment signal from the eye, we evaluated the RHT projection by injecting fluorescently labeled CTB into the eyes in 6- and 12-month-old APP/PS1 and wt mice. CTB is a highly sensitive retrograde neuroanatomical tracer, and using fluorescent Alexa Fluor conjugates of CTB, multiple neuroanatomical connections can be reliably studied and compared [15, 17]. We hypothesized that the RHT of APP/PS1 mice does not projected adequately to the SCN. As we expected and it is shown in Fig. 6a, we found intense CTB labeling of the SCN in 6- and 12-month-old wt mice; but little or no such labeling in the SCN in APP/PS1 mice at those ages. CTB staining was densitometric analyzed and results confirmed that reduction observed in the immunofluorescence images (Fig. 6b). These findings suggest that APP/PS1 mice have an important loss of retinal ganglion cell projections connecting in the hypothalamus.

As RHT pathway is altered in APP/PS1, we suggest that the information from retina to the hypothalamic areas is not incoming correctly, and consequently hypothalamic functionality is downregulated. Light signals reach the SCN via a direct projection from the retina through RHT using GABA as neurotransmitter, modulating circadian timekeeping [1, 2, 14]. It is synthesized from its immediate precursor glutamate by the rate-limiting enzyme GAD, which includes GAD65 and GAD67 forms [12]. Then, we used the anti-GAD67 antibody to identify GABAergic cells present within the hypothalamus, as previous described [65, 66]. We found GAD67 positive cells fairly uniform throughout the arcuate nucleus (Fig. 6c). Stereological analysis of GAD67 immunostaining revealed a significant decrease in both APP/PS1 and wt mice with aging (Fig. 6d). However, this reduction was higher in transgenic mice, and while no effects of genotype were observed for the protein expression of GAD67 in 6-month-old mice, GAD67 levels were significantly reduced in 12-month-old APP/PS1 mice compared to wt group (Fig. 6d). We confirmed these findings by western blot (Fig. 6e). GAD67 levels were significant reduced with age at hypothalamic level in both APP/PS1 and wt mice (Fig. 6f).

### Aβ accumulations and disturbances in the retinas of APP/PS1 mice

**Fig. 7:**
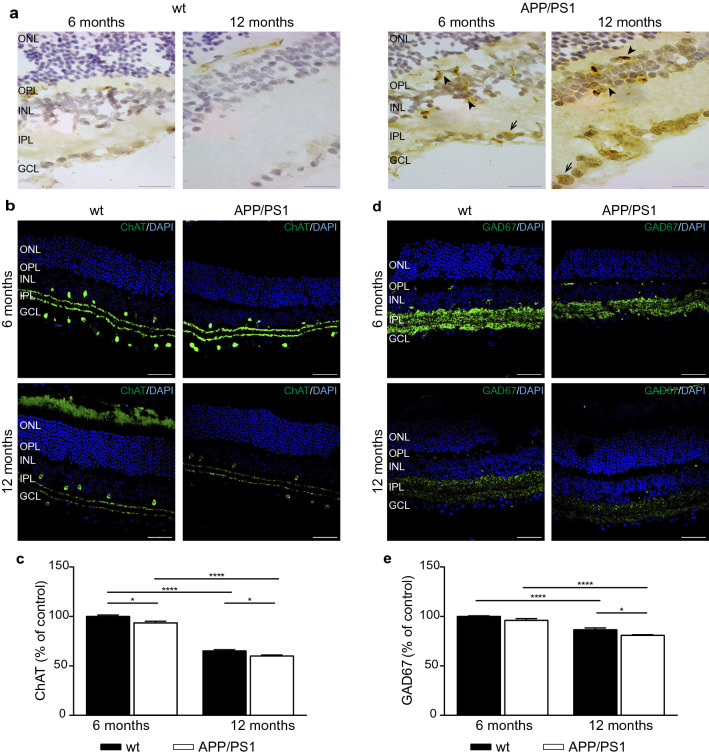
Morphological abnormalities in the retina of APP/PS1 mice. **a** Representatives images showing the immunoreactivity for Aβ (scale bar, 20 µm) in the retina of 6- and 12-month-old wt and APP/PS1 mice. In the wt retina, Aβ localizes to the cytoplasm of few RGCs (arrow), but in APP/PS1 mice, Aβ expression is markedly higher in RGCs (arrow), and is also clearly detectable in other neuronal populations (arrowhead). **b**, **c** Representative confocal images showing immunostaining of ChAT (scale bar, 50 µm) in the retina of wt and APP/PS1 mice and **c** quantitative analysis of the fluorescence intensity of ChAT staining (n = 3–5 mice per group). **d**, **e** Representative confocal images showing immunostaining of GAD67 (scale bar 50 µm) in the retina wt and APP/PS1 mice and **e** quantitative analysis of the fluorescence intensity of GAD67 staining (n = 3 mice per group). Nuclei are stained with DAPI (blue). Data are expressed as mean ± SD. *p < 0.05; ****p < 0.0001 using two-way ANOVA followed by Bonferroni’s multiple comparison post-test. *RGCs* retinal ganglion cells, *ChAT* choline acetyltransferase, *wt* wild type

Aβ deposition in the retina of APP/PS1 mice was previously found by Perez and colleagues [61]. Structurally, the neural retina contains several distinct layers: the inner limiting membrane (ILM), nerve fiber layer (NFL), ganglion cell layer (GCL), inner plexiform layer (IPL), inner nuclear layer (INL), outer plexiform layer (OPL), outer nuclear layer (ONL), outer limiting membrane (OLM), and the outermost photoreceptor layer (PRL). In our present study, most of the Aβ deposits were located in the INL, OPL, as well as in the GCL of the retina in APP/PS1 mice (Fig. 7a). These Aβ accumulation was already detected in 6 month-old APP/PS1 mice and are maintained at 12 months of age (Fig. 7a).

To examine whether Aβ deposits alters the retinal function of APP/PS1 mice, sections were immunostainned with cholinergic markers. ACh, one of the major excitatory neurotransmitter in the retina, is synthesized from choline by Acetyl Co-A by the enzyme choline acetyltransferase (ChAT). In the retina, the source of ACh are the starburst amacrine cells [22, 46]. We found robust ChAT immunoreactivity in amacrine cell bodies distributed in the INL and GCL and their processes in the IPL bands in 6-month-old wt mice (Fig. 7b). Similar patterns of immunoreactivity were observed in the 6-month-old APP/PS1 mouse retinas, but the labeling was less intense than in the wt mice (Fig. 7b). When we analyzed 12-month-old mice, ChAT immunoreactivity was significantly decreased in both mice groups, being greater this reduction in APP/PS1 mice compared with wt mice group. Stereological analysis of ChAT immunostaining confirmed these findings (Fig. 7c).

Starburst amacrine cells also contain the 67-kDa isoform of GAD [11]. GAD67 immunoreactivities were localized in amacrine cells in the INL and GCL, and in densely distributed immunoreactive processes and puncta in all laminae of the IPL (Fig. 7d). We observed lower GAD67 immunoreactivity in the IPL with age in both mice groups, but this reduction was also observed between APP/PS1 and wt mice at 12 months of age (Fig. 7d, e).

### Loss and morphological changes in melanopsin RGCs in APP/PS1 mice

**Fig. 8:**
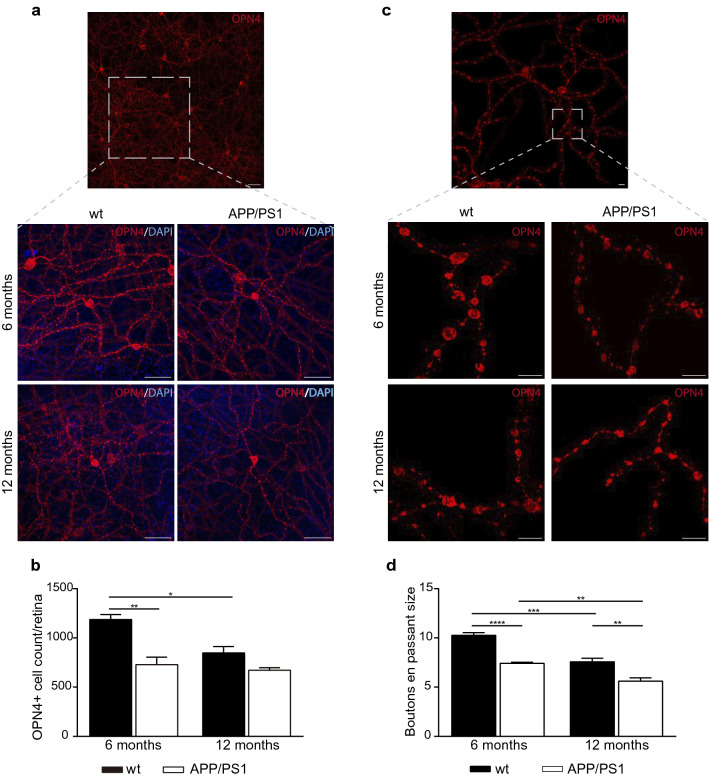
Morphological abnormalities in flat-mounted retinas of APP/PS1 mice. **a** Upper panel showing retinal area at low magnification, botom panels showing at higher magnification representative images of melanopsin immunoreactivity in the retina of wt and APP/PS1 mice (scale bar 50 µm). **b** The results of total analysis of retina showing reduced mRGC number in APP/PS1 mice compared to wt mice (n = 3–6 mice per group). **c** At high magnification (lower panels), melanopsin staining revealed abnormal morphology in the dendritic processes of mRGC with smaller *boutons en passant* in APP/PS1 mice (scale bar 10 µm). **d** Size analysis of these *boutons en passant* showed a significant reduced size in both 6- and 12-month-old APP/Ps1 mice compared to age-matched wt group. Note lower size of *boutons en passant* with age (n = 4 mice per group). Data are expressed as mean ± SD. **p < 0.01; ***p < 0.001; ****p < 0.0001 using two way ANOVA followed by Bonferroni’s multiple comparison post-test. *mRGCs* melanopsin retinal ganglion cells, *wt* wild type

As shown in Fig. 5, APP/PS1 mice have an important loss of retinal ganglion cell projections connecting in the hypothalamus. It was demonstrated that a set of ganglion cells belonging to the RTH expresses melanopsin [27, 29] and that these neurons can respond to light directly [10]. Here, we evaluated flat-mounted retinas from 6- and 12-month-old APP/PS1 and wt mice to evaluate total account of mRGCs. After immunostaining with an antibody targeting melanopsin (OPN4), we found that the mRGCs account was significantly reduced in 6-month-old APP/PS1 compared with age-matched wt mice (Fig. 8a, b). This decrease in the mRGCs account was also observed with the age in wt mice, but not in transgenic mice (Fig. 8a, b).

We also analyzed mRGCs morphology investigating characteristic varicosities present in the dendritic processes. Melanopsin was accumulated in the dendritic processes and in the *boutons en passant* characteristics of the mRGCs (Fig. 8c). Detailed examination of the reconstructed arbors provided insight into the structure of these boutons. The most striking changes between APP/PS1 and wt mice were in the size of these bouton-like varicosities along the axon terminal (Fig. 8c). The average bouton area was decreased with age in both mice groups, however the most interesting results were the reduced bouton size an early event in the retina from APP/PS1 when compared with age-matched wt mice as the melanopsin marker was found significantly reduced at 6-month-old and such levels were maintained with aging (Fig. 8d). Taken together, our findings demonstrate the abnormal number and morphology of mRGCs in APP/PS1 mice, and could be considered an early detectable event.

### Retinal functional deficits in APP/PS1 mice

**Fig. 9:**
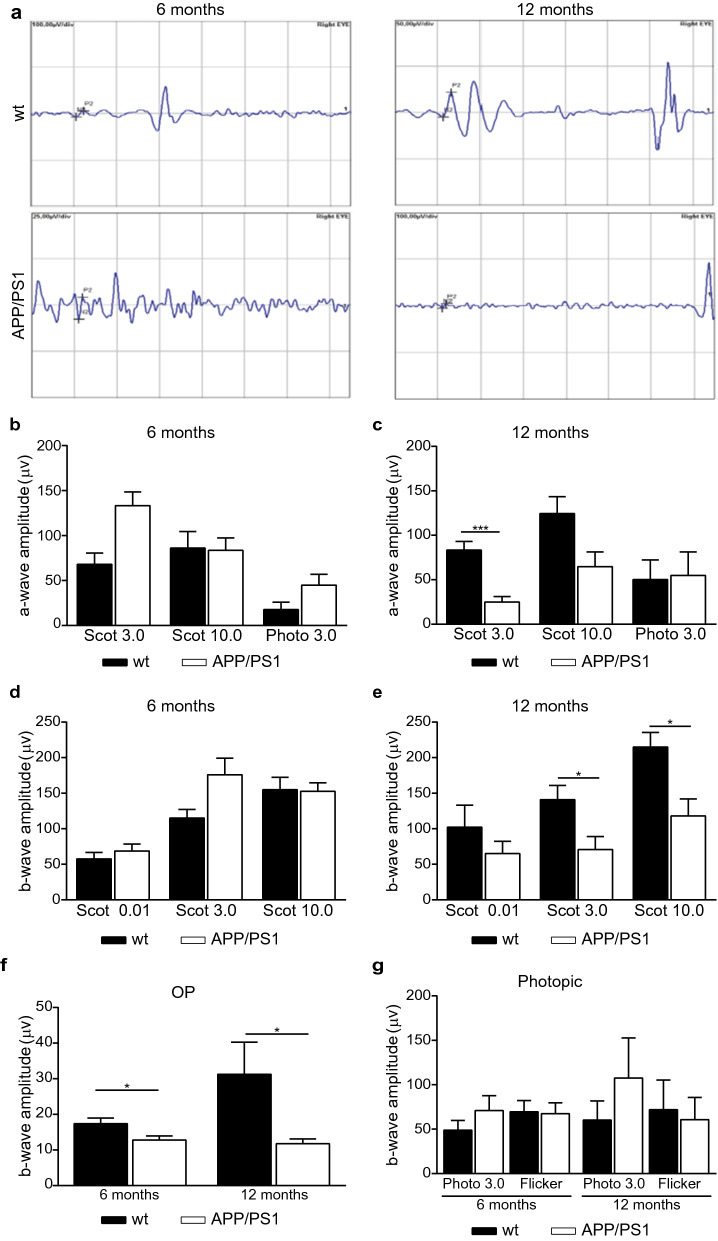
Retinal functional alterations in 6- and 12-month-old wt and APP/PS1 mice. **a** Representative *a*- and *b*-waveforms of scotopic ERG recordings obtained from 6- to 12-month-old mice. **b**, **c** Quantitative analysis of amplitudes of *a-*waves in 6- and 12-months-old APP/PS1 and wt mice. **d**, **e** Quantitative analysis of amplitudes of *b* waves in 6- and 12-month-old APP/PS1 and wt mice. **f** Quantitative analysis of oscillatory potentials under scotophic conditions in 6- and 12-month-old APP/PS1 and wt mice. **g** Quantitative analysis of photophic ERG recorded from 6- to 12-month-old APP/PS1 and wt mice. Data are expressed as mean ± SEM. *p < 0.05; ***p < 0.001 using *t*-test (n = 6–9 mice per group). *Scot 0.01* Scotopic test 0.01 cds/m^2^, *Scot 3.0* Scotopic test 3.0 cds/m^2^, *Scot 10.0* Scotopic test 10.0 cds/m^2^, *OP* Oscillatory potentials, *Photo 3.0* Photopic test 3.0 cds/m^2^, *wt* wild type

To evaluate the functional integrity of the retinas of APP/PS1 mice, we performed ERG testing with 6- and 12-month-old APP/PS1 and age-matched wt mice, which provides a functional measure of various neuronal cell types. The electrical activity of rod and cone photoreceptors, in response to light stimulation, translates into the ERG *a*-wave, while other neurons contribute to the ERG *b*-wave generated by the inner retinal components. When comparing *a*-wave response in 6-month-old APP/PS1 mice compared to wt mice in scotopic conditions, we found no significant differences in scotopic conditions (Fig. 9a, b). However, at the age of 12 months, APP/PS1 mice *a*-wave amplitude was significantly decreased in APP/PS1 compared to the wt mice under 3.00 cds/m^2^ flash intensity (Fig. 9a, c). Concerning the inner retinal response, as assessed by the *b*-wave amplitude measurement corresponding mainly to the activity of the bipolar neurons, significant differences were observed in *b*-amplitudes from APP/PS1 and wt mice aging 6 and 12-months under scotopic condition. At 6 months, differences in ERG recordings were found only under 3.00 cds/m^2^ stimulation to analyze oscillatory potentials using 0.067 Hz frequency (Fig. 9f). The amplitudes of *b*-waves were significantly decreased in 12-month-old APP/PS1 mice compared to wt mice when tested at 10.0 cds/m^2^, and at 3.00 cds/m^2^ intensities (Fig. 9a, e). Oscillatory potentials responses were also significantly reduced in 12-month-old APP/PS1 mice compared to age-matched wt mice (Fig. 9f). Under standard lighting conditions used to assess the cone photoreceptor response, the photopic ERG measurements showed no significant difference between APP/PS1 and wt mice (Fig. 9g). Altogether, these data indicate that APP/PS1 retina present a diminished response, mainly at the inner retinal neurons (mainly bipolar cells) which is already detectable at the age of 6 months, but is enhanced at 12 months, corresponding with advanced-stage of pathology.

## Discussion

In the present study, we analysed the circadian rhythm in 6- and 12-month-old APP/PS1 mice, and the retinal pathway involved in its control. We show altered circadian clock genes expression not only in the hypothalamus but also in two extra-hypothalamic brain regions, cerebral cortex and hippocampus, in APP/PS1 mice. These alterations were observed in 6-month-old transgenic mice and were exacerbated at 12 months, corresponding with more advanced stage of pathology. Our findings support the hypothesis that Aβ-related pathology is associated with changed expression of circadian clock genes [52]. In addition to altered expression and rhythmicity of clock genes, we found reduced RHT projections in the SCN of APP/PS1 mice, pointing that circadian clock alterations we observed in this AD mouse model may be due, at least in part, to a deficiency of an entrainment signal from the eye. Consequently, hypothalamic GABAergic response was downregulated in APP/PS1 mice, which would explain the disrupted circadian rhythmicity in these transgenic AD mice, based on its major role in circadian control [1]. Furthermore, retinal Aβ accumulation also evidences retinal dysfunction in AD [39]. Here, we showed Aβ deposits with reduced ChAT levels, indicating lower availability of the excitatory neurotransmitter ACh, and GABAergic cells in the retina of APP/PS1 mice. Additionally, we reported the abnormal number and morphology of mRGCs as an early event in the retina of APP/PS1 mice. Finally, our data also demonstrate Aβ-related disturbance in retinal neuronal transmission as indicated by defective ERG activity.

The circadian rhythm consists of an autoregulatory positive and negative feedback transcriptional network, including positive regulators (*Clock* and *Arntl*) that activate the transcription of the Period (*Per1*, *Per2* and *Per3*), Cryptochrome (*Cry1* and *Cry2*) genes, the negative regulators. We found that the RNA expression of clock genes are disrupted mainly in the hypothalamus in the 6-month-old APP/PS1 mice compared with age-matched wt mice, whereas in the two extra-hypothalamic brain areas, hippocampus and cerebral cortex, these alterations were less severe. In the hypothalamus, the most remarkable result was the disruption of circadian rhythmicity of negative regulators *Cry1, Cry2 Per1*, and *Per2* in 6-month-old APP/PS1 mice. The hypothesis of a single central clock has been challenged by the discovery of circadian oscillation in other extra-hypothalamic brain regions, including hippocampus and cerebral cortex [24, 43]. In our study, and in contrast to hypothalamic oscillation, clock gene rhythmicity is maintained in hippocampus and cerebral cortex in 6-month-old APP/PS1 mice. Our findings agree with other studies where, hippocampal clock gene expression was not different between young APP/PS1 mice and age-matched wt mice [59]. However, hippocampal oscillation is lost in aged APP/PS1 with altered expression patterns of core clock genes similar to those previously published in similar aged APP/PS1 mice [44].

A number of other alterations in the circadian pattern of activity of APPS/PS1 mice were detected across several parameters of rhythmicity. Notably, there is a delayed expression peak (acrophase) of hypothalamic *Arntl* transcription in both 6- and 12-month-old transgenic mice compared with age-matched wt mice. Similar circadian deregulation of *Arntl* was reported in AD patients’ fibroblasts [18]. It is important to note that *Arntl* not only regulates the transcription of core clock genes, but also modulates other important physiological functions [54]. In parallel, it was reported that Aβ could induced *Arntl* degradation [63]. As we have demonstrated Aβ accumulation in hypothalamic neurons, corroborating AD-related morphological alterations in hypothalamus [6], we support the robust connection between altered expression of circadian clock genes and Aβ accumulation in the hypothalamus affecting the control of biological functions exercised by this brain area.

The RHT connects retina with the SCN by photic information to synchronize endogenous circadian rhythms with the light–dark cycle. RHT projections consists of retinal ganglion cell axons that project directly to the SCN [49]. Stimulation of the SCN via mediation by RHT initiates a cascade of transcriptional–translational feedback loops that eventually lead circadian rhythms. Since RHT-projecting retinal ganglion cell axons in the SCN are significantly reduced in APP/PS1, this dysfunctional pathway would result in reduced hypothalamic activity.

Here we found a significant decrease in GAD expression in both APP/PS1 and wt mice with aging, being more severe in transgenic mice. GAD catalyzes the production of GABA from glutamate [12], and exists as two isoforms with molecular masses of 65 and 67 kDa (GAD65 and GAD67, respectively), which are encoded by independent genes [20]. The expression of both *Gad65* and *Gad67* mRNA in POMC neurons is consistent with the previous reports of GABA release from POMC neurons in primary cultures and hypothalamic slice preparations [30, 31]. The dysfunction of GABAergic pathways and the loss of GABAergic neurons are strongly implicated in AD pathogenesis. Thus, our findings may indicate a reduction in the SCN GABA content with its consequent effects on the regulation of the circadian rhythm in the SCN, as has been proposed [58]. This hypothesis is also supported by a recent study performed in mice suggested that inhibition via retinal GABAergic input alters the light sensitivity of the circadian clock [64].

The pathology of AD also shows neurodegeneration in retinal and optic nerve tissues. Aβ deposits have been reported in the retina of AD patients and AD transgenic mice [19, 40, 61]. Here, we confirmed in APP/PS1 mice the presence of Aβ deposition in retina, replicating and expanding previous findings of intracellular and small extracellular deposits of Aβ in APP/PS1 retina. Aβ deposits in retina initiate a cascade of pathological events creating a toxic microenvironment leading to retinal degeneration [4]. In our present study, we found a reduction in the retinal levels of ChAT with aging but also in APP/PS1 mice compared with wt mice. This is an enzyme involved in the synthesis of ACh, one of the major excitatory neurotransmitter in the retina [25]. We propose this ChAT reduction as a direct effect of Aβ deposition in retina in transgenic mice. This is supported by the observation that ChAT activity is reduced significantly when neurons are exposed to Aβ aggregates [23] or soluble Aβ oligomers [56].

Starburst amacrine cells process complex visual signals in the retina using the both classical neurotransmitters, ACh and GABA [41, 45]. Indeed, starburst amacrine cells are the only cholinergic neurons in the retina [21, 70]. We also found a similar expression pattern of GAD with lower immunoreactivity with aging, but this reduction more pronounced in APP/PS1 mice. Since, ACh and GABA were co-released from Starburst amacrine cells to mediate fast synaptic transmission, we suggest that process of light signals is seriously compromised in AD retina.

We also found mRGCs loss in APP/PS1 mice accompanied by morphological changes affecting melanopsin distribution in dendrites and boutons, similarly than that observed in AD retinas [40]. This loss of mRGCs may be explained as a retrograde axonal degeneration derived from pathology of SCN through the RTH, as previously proposed [40]. However, in the present study there does not seem to be a loss of neurons in the hypothalamic region in APP/PS1 mice (Fig. 5h). Thus, we propose that the presence of Aβ in retina may exert a direct toxic effect on cell viability and functionality. Supporting this last theory, ERG dysfunction was reported in 12 to 16 month-old APP/PS1 mice, most likely the result of Aβ deposition in the retina of these mice [61]. More recently, functional retinal alterations have been reported in 3 month old 5xFAD mice [48]. Consistent with the mentioned studies, we demonstrate here that ERG dysfunction is already manifested at early age in APP/PS1 mice. These quantitative electrophysiological findings expand our understanding of early retinal functional changes that may precede cognitive decline in AD [39, 50]. Retinal ganglion cell dysfunction, as detected by ERG, may be a clinically useful, non-invasive in vivo biomarker for early disease detection, as has been recently proposed [3]. In summary, our findings support the role of retina in AD pathology showing early AD-like pathological alterations and contributing to the circadian rhythm dysfunction.

## Supplementary Information

**Additional file 1: Table S1.** Sequence of primers used for qRT-PCR studies.

**Additional file 2: Table S2.** Circadian parameters for clock gene expression in mice hypothalamus.

**Additional file 3: Table S3.** Circadian parameters for clock gene expression in mice hippocampus.

**Additional file 4: Table S4.** Circadian parameters for clock gene expression in mice cerebral cortex.

## Data Availability

Data is contained within the article. There are no novel reagents or materials for others to request.
