# Supplementary material for: Disturbed circadian rhythm and retinal degeneration in a mouse model of Alzheimer’s disease
Source: Acta Neuropathol Commun. 2023 Mar 31;11:55. doi: 10.1186/s40478-023-01529-6 (PMC10067208; doi:10.1186/s40478-023-01529-6)
Supplement: Supplementary file 1 — Additional file 1: Table S1. Sequence of primers used for qRT-PCR studies. [file 40478_2023_1529_MOESM1_ESM.docx]

Supplementary Table 1. Sequence of primers used for qRT-PCR studies.

| Gene |  | Sequence (5´- 3´) |
| --- | --- | --- |
| *Circadian locomotor output cycles kaput (Clock)* | Fw | CCTTGCGTCTGTGGGTGTT |
|  | Rv | TGTCATCTTCTTCCACCAATCCA |
| *Aryl hydrocarbon receptor nuclear translocator-like (Arntl)* | Fw | CGCCTCTACCTGTTCAAAGAAAAA |
|  | Rv | TCACCCGTATTTCCCCGTTC |
| *Period circadian clock 1 (Per1)* | Fw | AAACGGCAAGCGGATGG |
|  | Rv | GCCATACAGTGGAGGACGAAA |
| *Period circadian clock 2 (Per2)* | Fw | CAGAGGAGAAGACTCCGCAC |
|  | Rv | TTGCTGTCGCTGGATGATGT |
| *Period circadian clock 3 (Per3)* | Fw | ACGAAGCTCCTCGAATCCCT |
|  | Rv | GAATCTGACGGGCGAGTGTT |
| *Cryptochrome 1 (Cry1)* | Fw | CGGTAGAGGAAGTCGGGGTG |
|  | Rv | AGACACTGAAGCAAAAATCGCC |
| *Cryptochrome 2 (Cry2)* | Fw | TGAAGAACTGACCAAGCGGG |
|  | Rv | GTTGGTGATTGGCTTCTCTGC |
| *Hypoxanthine-guanine phosphoribosyltransferase (Hprt)* | Fw | GTTGGGCTTACCTCACTGCT |
|  | Rv | TAATCACGACGCTGGGACTG |
