# Supplementary material for: Disturbed circadian rhythm and retinal degeneration in a mouse model of Alzheimer’s disease
Source: Acta Neuropathol Commun. 2023 Mar 31;11:55. doi: 10.1186/s40478-023-01529-6 (PMC10067208; doi:10.1186/s40478-023-01529-6)
Supplement: Supplementary file 2 — Additional file 2: Table S2. Circadian parameters for clock gene expression in mice hypothalamus. [file 40478_2023_1529_MOESM2_ESM.docx]

Supplementary Table 2. Circadian parameters for clock gene expression in mice hypothalamus.

| Gene | Age (months) | Genotype | Period (h) | Phase (h) | Amplitude | *p*-value |
| --- | --- | --- | --- | --- | --- | --- |
| *Clock* | 6 | wt | 24.00 | 23.37 | 0.11 | 0.12 |
|  |  | APP/PS1 | 24.00 | 3.82 | 0.13 | 0.08 |
|  | 12 | wt | 24.00 | 18.21 | 0.06 | 0.49 |
|  |  | APP/PS1 | 24.00 | 4.14 | 0.13 | **0.03** |
| *Arntl* | 6 | wt | 24.00 | 23.75 | 0.23 | **7e-3** |
|  |  | APP/PS1 | 24.00 | 2.93 | 0.38 | **5.13e-4** |
|  | 12 | wt | 24.00 | 0.56 | 0.17 | **0.03** |
|  |  | APP/PS1 | 24.00 | 3.41 | 0.22 | 0.06 |
| *Cry1* | 6 | wt | 24.00 | 19.26 | 0.35 | **8.36e-4** |
|  |  | APP/PS1 | 24.00 | 20.20 | 0.10 | 0.26 |
|  | 12 | wt | 24.00 | 10.27 | 0.01 | 0.93 |
|  |  | APP/PS1 | 24.00 | 21.16 | 0.17 | 0.19 |
| *Cry2* | 6 | wt | 24.00 | 22.01 | 0.21 | **0.04** |
|  |  | APP/PS1 | 24.00 | 2.01 | 0.14 | 0.15 |
|  | 12 | wt | 24.00 | 1.20 | 0.08 | 0.51 |
|  |  | APP/PS1 | 24.00 | 6.47 | 0.44 | **1e-3** |
| *Per1* | 6 | wt | 24.00 | 21.87 | 0.26 | 0.06 |
|  |  | APP/PS1 | 24.00 | 3.33 | 0.07 | 0.44 |
|  | 12 | wt | 24.00 | 2.18 | 0.16 | 0.24 |
|  |  | APP/PS1 | 24.00 | 5.41 | 0.51 | 0.06 |
| *Per2* | 6 | wt | 24.00 | 20.76 | 0.24 | **0.02** |
|  |  | APP/PS1 | 24.00 | 13.08 | 0.20 | 0.09 |
|  | 12 | wt | 24.00 | 8.62 | 0.18 | 0.36 |
|  |  | APP/PS1 | 24.00 | 12.13 | 0.19 | 0.12 |
| *Per3* | 6 | wt | 24.00 | 18.77 | 0.34 | **2e-3** |
|  |  | APP/PS1 | 24.00 | 17.17 | 0.17 | 0.09 |
|  | 12 | wt | 24.00 | 9.84 | 0.23 | 0.15 |
|  |  | APP/PS1 | 24.00 | 11.12 | 0.28 | **5e-3** |
| wt: wild type; h: hour. | | | | | | |
