# Supplementary material for: Disturbed circadian rhythm and retinal degeneration in a mouse model of Alzheimer’s disease
Source: Acta Neuropathol Commun. 2023 Mar 31;11:55. doi: 10.1186/s40478-023-01529-6 (PMC10067208; doi:10.1186/s40478-023-01529-6)
Supplement: Supplementary file 3 — Additional file 3: Table S3. Circadian parameters for clock gene expression in mice hippocampus. [file 40478_2023_1529_MOESM3_ESM.docx]

Supplementary Table 3. Circadian parameters for clock gene expression in mice hippocampus.

| Gene | Age (months) | Genotype | Period (h) | Phase (h) | Amplitude | *p*-value |
| --- | --- | --- | --- | --- | --- | --- |
| *Clock* | 6 | wt | 24.00 | 22.52 | 0.13 | 0.11 |
|  |  | APP/PS1 | 24.00 | 8.35 | 1e-3 | 0.98 |
|  | 12 | wt | 24.00 | 18.86 | 0.21 | 0.12 |
|  |  | APP/PS1 | 24.00 | 0.94 | 0.12 | 0.12 |
| *Arntl* | 6 | wt | 24.00 | 1.59 | 0.18 | **0.03** |
|  |  | APP/PS1 | 24.00 | 4.54 | 0.10 | 0.16 |
|  | 12 | wt | 24.00 | 20.82 | 0.19 | 0.08 |
|  |  | APP/PS1 | 24.00 | 0.21 | 0.22 | **0.02** |
| *Cry1* | 6 | wt | 24.00 | 19.86 | 0.30 | **3e-3** |
|  |  | APP/PS1 | 24.00 | 19.26 | 0.40 | **9.4e-4** |
|  | 12 | wt | 24.00 | 17.91 | 0.56 | **0.03** |
|  |  | APP/PS1 | 24.00 | 17.75 | 0.16 | 0.24 |
| *Cry2* | 6 | wt | 24.00 | 17.69 | 0.25 | **0.02** |
|  |  | APP/PS1 | 24.00 | 18.11 | 0.36 | **6.57e-3** |
|  | 12 | wt | 24.00 | 19.04 | 0.31 | 0.11 |
|  |  | APP/PS1 | 24.00 | 5.93 | 0.33 | 0.07 |
| *Per1* | 6 | wt | 24.00 | 18.98 | 0.20 | 0.06 |
|  |  | APP/PS1 | 24.00 | 19.18 | 0.24 | **0.02** |
|  | 12 | wt | 24.00 | 18.08 | 0.38 | 0.11 |
|  |  | APP/PS1 | 24.00 | 6.31 | 0.08 | 0.47 |
| *Per2* | 6 | wt | 24.00 | 17.83 | 0.46 | **3.7e-4** |
|  |  | APP/PS1 | 24.00 | 17.91 | 0.28 | 0.12 |
|  | 12 | wt | 24.00 | 16.62 | 0.57 | **0.03** |
|  |  | APP/PS1 | 24.00 | 18.80 | 0.26 | 0.08 |
| *Per3* | 6 | wt | 24.00 | 17.77 | 0.40 | **3e-3** |
|  |  | APP/PS1 | 24.00 | 19.40 | 0.44 | **3e-3** |
|  | 12 | wt | 24.00 | 18.25 | 0.44 | **0.04** |
|  |  | APP/PS1 | 24.00 | 21.43 | 0.11 | 0.45 |

wt: wild type; h: hour.
