# Supplementary material for: Disturbed circadian rhythm and retinal degeneration in a mouse model of Alzheimer’s disease
Source: Acta Neuropathol Commun. 2023 Mar 31;11:55. doi: 10.1186/s40478-023-01529-6 (PMC10067208; doi:10.1186/s40478-023-01529-6)
Supplement: Supplementary file 4 — Additional file 4: Table S4. Circadian parameters for clock gene expression in mice cerebral cortex. [file 40478_2023_1529_MOESM4_ESM.docx]

Supplementary Table 4. Circadian parameters for clock gene expression in mice cerebral cortex.

| Gene | Age (months) | Genotype | Period (h) | Phase (h) | Amplitude | *p*-value |
| --- | --- | --- | --- | --- | --- | --- |
| *Clock* | 6 | wt | 24.00 | 21.00 | 0.11 | **0.04** |
|  |  | APP/PS1 | 24.00 | 3.29 | 0.02 | 0.60 |
|  | 12 | wt | 24.00 | 13.18 | 0.04 | 0.42 |
|  |  | APP/PS1 | 24.00 | 21.96 | 0.15 | 0.11 |
| *Arntl* | 6 | wt | 24.00 | 2.09 | 0.14 | **6e-3** |
|  |  | APP/PS1 | 24.00 | 4.65 | 0.14 | **0.03** |
|  | 12 | wt | 24.00 | 4.93 | 0.12 | 0.27 |
|  |  | APP/PS1 | 24.00 | 21.78 | 0.36 | **7e-3** |
| *Cry1* | 6 | wt | 24.00 | 17.85 | 0.47 | **7.47e-4** |
|  |  | APP/PS1 | 24.00 | 19.25 | 0.19 | 0.12 |
|  | 12 | wt | 24.00 | 13.85 | 0.24 | 0.16 |
|  |  | APP/PS1 | 24.00 | 20.90 | 0.71 | **3.04e-4** |
| *Cry2* | 6 | wt | 24.00 | 17.74 | 0.47 | **3.78e-4** |
|  |  | APP/PS1 | 24.00 | 17.67 | 0.17 | **0.04** |
|  | 12 | wt | 24.00 | 13.80 | 0.20 | 0.18 |
|  |  | APP/PS1 | 24.00 | 20.68 | 0.58 | **3e-3** |
| *Per1* | 6 | wt | 24.00 | 17.99 | 0.46 | **3e-3** |
|  |  | APP/PS1 | 24.00 | 16.53 | 0.07 | 0.38 |
|  | 12 | wt | 24.00 | 5.67 | 0.23 | 0.05 |
|  |  | APP/PS1 | 24.00 | 21.38 | 0.31 | **0.01** |
| *Per2* | 6 | wt | 24.00 | 17.98 | 0.54 | **1.98e-4** |
|  |  | APP/PS1 | 24.00 | 19.25 | 0.21 | **0.03** |
|  | 12 | wt | 24.00 | 15.61 | 0.14 | 0.37 |
|  |  | APP/PS1 | 24.00 | 19.05 | 0.73 | **1e-3** |
| *Per3* | 6 | wt | 24.00 | 17.43 | 0.52 | **2.37e-5** |
|  |  | APP/PS1 | 24.00 | 18.12 | 0.20 | 0.09 |
|  | 12 | wt | 24.00 | 15.10 | 0.44 | **0.03** |
|  |  | APP/PS1 | 24.00 | 20.20 | 0.69 | **7e-3** |

wt: wild type; h: hour.
